# Application of bimolecular fluorescence complementation in studying the topology of polytopic proteins

**DOI:** 10.1186/1750-1326-7-S1-O8

**Published:** 2012-02-07

**Authors:** Xingya Chang, Fei Dou

**Affiliations:** 1 Key Laboratory of Cell Proliferation and Regulation Biology, Ministry of Education, College of Life Science, Beijing Normal University, Beijing100875, China

## Background

Bimolecular Fluorescence Complementation (BiFC) assay has proven very useful to detect protein-protein interaction, protein-DNA/RNA interaction and protein-ligand interaction under physiological condition or near-physiological condition, even the interaction is too weak or too transient to be detected by other assays such as Co-IP or Y2H. Here, instead of studying two proteins interaction, we report a novel, intramolecular application of BiFC in studying the topology of proteins with multi transmembrane domains. Polytopic protein Presenilin 1 (PS1) is a critical component of the gamma-secretase complex that is responsible for the intramembranous cleavage of several type I transmembrane proteins. Although essential for a correct understanding of structure-function relationships, its exact topology used to be an issue of strong controversy. According to the Kyte-Doolittle plot, PS1 has ten hydrophobic regions (HR) potentially to be alpha-helical transmembrane domains. Mature PS1 protein undergoes endoproteolysis resulting in stable NH2- and COOH-terminal fragments (PS1-NTF and -CTF). All published models agree that the first six HR cross the membrane, implying a consensus for the topology of the PS1-NTF. In contrast, the localization of the last HR caused the most diversity. Most topology models for PS1, especially studies carried out on SEL-12, place its HR 10 in the cytosolic space. However, several recent observations suggest that HR10 may be integrated into the membrane.

## Method

In the experiments presented here, we have investigated the topology of PS1-CTF by using BiFC. The fluorescent protein Venus was split at 173aa to generate two fragments, the NH2-terminal fragment of Venus (VN) and the COOH-terminal fragment of Venus (VC). The VN and VC fragments were fused to the both ends of the test protein respectively. Our hypothesis is that if the test protein span the membrane odd times, due to the barrier of lipid bilayer, the two fragments of Venus are not able to reach each other to accomplish the reconstitution, hence no fluorescent signal will be observed; on the other hand, when the test protein is with even times trans-membrane domains, the VN and VC have the opportunity to be brought together and the fluorescent signal will be detected.

## Results and conclusion

To test whether our hypothesis is feasible, we first tested it by verifying the six transmembrane topology of PS1-NTF. Coding fragment for all the 6 transmembrane domains of PS1-NTF has been fused with VN and VC at both ends respectively. When such a fusion protein expressed in COS7 cells, as we expected, the reconstituted fluorescent signal could be detected in the cells. Same results were observed when the first 2 or 4 HRs of PS1-NTF was fusion expressed with VN and VC. No fluorescent signal was detected when the first 5 HRs were inserted between VN and VC. Similar results were got when the first HR or 3 HRs were fusion expressed with VN and VC. The above experimental results imply that our model is practical and suitable to study the topology of multi trans-membrane proteins. Next, we applied the same strategy to study the topology of PS1-CTF. A plasmid was generated to express VN-PS1CTF-VC fusion protein. This fusion protein contains the last 3 HRs of PS1 (HR8-10). If the 10^th^ HR, whose localization is controversy, stays in the cytosol, then we should see the reconstituted fluorescent signal; if the 10^th^ HR is a transmembrane segment, there will be no signal. The result from this experiment shown there is no detectable fluorescence, which indicate the N-terminus and C-terminus of PS1-CTF locates on the different sides of cell membrane, hence the 10^th^ HR of PS1 is a transmembrane segment. As a control, when the VN-HR(9-10)-VC expressed in COS cells, strong fluorescence can be detected. Furthermore, there is no significant fluorescence in COS cells express VN-HR10-VC, which support the model that 10^th^ HR is a transmembrane domain rather than a cytosolic domain. As it’s well known that the crystal structure of polytopic protein is harder to get than cytosolic protein or protein with single transmembrane domain, but determine the correct topology of such proteins is important for the understanding of downstream signal transduction and drug development. Our study provides a simple method to acquire first-hand topological information on such issue.

